# Editorial: Current Aspects in Chemopreventive Strategies

**DOI:** 10.3389/fphar.2020.607503

**Published:** 2021-01-04

**Authors:** Hardeep Singh Tuli, Mukerrem Betul Yerer, Katrin Sak

**Affiliations:** ^1^ Department of Biotechnology, Maharishi Markandeshwar (Deemed to be University), Ambala, India; ^2^ Faculty of Pharmacy Department of Pharmacology, Erciyes University Drug Application and Research Center, Erciyes University, Kayseri, Turkey; ^3^ NGO Praeventio, Tartu, Estonia

**Keywords:** chemopreventive agents, targeted therapies, personalized medicine, antimutagenic, neoadjuvant therapy

**Editorial on the Research Topic**

**Current Aspects in Chemopreventive Strategies**

Despite extensive studies, cancer remains one of the most dreadful diagnoses and biggest challenges for human health all over the world, representing a leading cause of death in the industrialized countries. Various chemotherapeutic drugs, such as Doxorubicin, Tamoxifen and Paclitaxel, have been used for the treatment of tumours for more than half a century; however, there are still no curative options currently available in clinical settings and the severe adverse effects of these drugs threaten the well-being of the patients seriously. Current evidence suggests that further knowledge is urgently needed to clarify the unknown properties and molecular mechanisms of action of various chemopreventive molecules. This special issue attempts to highlight the ongoing advancement in chemopreventive and therapeutical approaches, in the context of cancer prevention and therapy. In particular, the specific objective of this collection was to gather the results of well-designed *in silico*, *in vitro* and *in vivo* preclinical studies, to draw scientists’ attention towards precision and personalized medicine in cancer patients by performing targeted therapies. This issue is a collection of eleven articles that have beautifully described chemopreventive approaches with strong therapeutic applications. In this special issue you can find some papers on novel drugs such as a new class of magnetite (Fe3O4) particles, coined as “Single Crystalline Micrometric Iron Oxide Particles” (SCMIOPs), which is obtained by hydrothermal synthesis and were tested for their cytotoxic effects on different melanoma types. Furthermore, you can find the results of *in vitro* antitumor activity of some novel styryllactones, a class of compounds obtained from the genus Goniothalamus (Annonaceae). In addition to these novel compound studies with anticancer activity, on the basis of personalized medicine applications Liu et al has conducted a meta-analyses to determine the association between genetic polymorphisms and platinum-induced toxicity which summarizes the pharmacogenomic reports that focused on commonly reported platinum.

Additionally, our Italian colleagues supervised by Dr. Lenzi clearly demonstrated the antimutagenic activities of a natural bioactive compound, 6-MITC, on human lymphoblastic cells. An interesting paper of Calcabrini et al reported the chemopotentiation of two frequently used conventional drugs, Doxorubicin and Cisplatin, by a well-known phytochemical sulforaphane, presenting a possibility to mitigate the toxicity associated with the use of these chemotherapeutics. Moreover, the team of Yu et al demonstrated the antimetastatic effects of metapristone on breast cancer cells co-incubated with HPMEC, by interfering with the adhesion-invasion processes. With prospects to be translated in clinical practice in the future, Desai et al thoroughly summarized the recent advancements of nanotechnology-based chemopreventive strategies. Last but not least, the Indian clinical oncologists presented in their contribution a strong basis for further developing the Indian guidelines to prevent and manage chemotherapy-induced nausea and vomiting. Bhatia et al. conducted a study to reveal the antioxidant potential and DNA protective abilities of *R. cinerea*. In addition, antiproliferative and apoptosis induction potential against immortalized L6 cell line have also discussed by the authors. In another study, Chan et al. suggested the potential of HLJDD as a neoadjuvant therapy to minimize chemo toxicity effects by reducing diarrhoea and improving tumour response. A review on anticancer potential of BC and its components to treat gastrointestinal diseases and distinctive cancer types, is another highlight of this special issue. We hope that you all will enjoy the reading of this thematic issue on **“Current Aspects in Chemopreventive Strategies”** from Frontiers in Pharmacology.

